# Co-interviewing across gender and culture: expanding qualitative research methods in Melanesia

**DOI:** 10.1186/1471-2458-14-922

**Published:** 2014-09-06

**Authors:** Michelle L Redman-MacLaren, Unia K Api, Matupit Darius, Rachael Tommbe, Tracie A Mafile’o, David J MacLaren

**Affiliations:** College of Medicine and Dentistry, James Cook University, McGregor Rd, Smithfield, Cairns, Australia; Department of Research and Postgraduate Studies, Pacific Adventist University, Port Moresby, National Capital District, Papua New Guinea; School of Theology, Pacific Adventist University, Port Moresby, National Capital District, Papua New Guinea; School of Health Science, Pacific Adventist University, Port Moresby, National Capital District, Papua New Guinea; Deputy Vice Chancellor, Pacific Adventist University, Port Moresby, National Capital District, Papua New Guinea

**Keywords:** Co-interviewing, Qualitative research, Public health research, Culture, Gender, Power, Papua New Guinea, Melanesia

## Abstract

**Background:**

The social and cultural positions of both researchers and research participants influence qualitative methods and study findings. In Papua New Guinea (PNG), as in other contexts, gender is a key organising characteristic and needs to be central to the design and conduct of research. The colonial history between researcher and participant is also critical to understanding potential power differences. This is particularly relevant to public health research, much of which has emerged from a positivist paradigm. This paper describes our critical reflection of flexible researcher responses enacted during qualitative research in PNG.

**Methods:**

Led by a senior male HIV researcher from PNG, a male from a PNG university and a female from an Australian university conducted qualitative interviews about faith-based responses to HIV in PNG. The two researchers planned to conduct one-on-one interviews matching gender of participants and interviewer. However, while conducting the study, four participants explicitly requested to be interviewed by both researchers. This experience led us to critically consider socially and culturally situated ways of understanding semi-structured interviewing for public health research in Melanesia.

**Results:**

New understandings about public health research include: (i) a challenge to the convention that the researcher holds more power than the research participant, (ii) the importance of audience in Melanesia, (iii) cultural safety can be provided when two people co-interview and (iv) the effect an esteemed leader heading the research may have on people’s willingness to participate. Researchers who occupy insider-outsider roles in PNG may provide participants new possibilities to communicate key ideas.

**Conclusions:**

Our recent experience has taught us public health research methods that are gender sensitive and culturally situated are pivotal to successful research in Melanesia. Qualitative research requires adaptability and reflexivity. Public health research methods must continue to expand to reflect the diverse worldviews of research participants. Researchers need to remain open to new possibilities for learning.

## Background

The social, cultural and professional positions of both researcher and research participant influence qualitative methods and study findings. When conducting interviews for public health research about sexual health in Papua New Guinea (PNG) and Melanesia more broadly, it has been our practice to match the gender of the research participant with the researcher [1–5]. This gendered approach is consistent with many cultural practices and obligations in Melanesia and is also informed by feminist and decolonising research theory which explains research as a power-laded process [6–8]. We understand that having power means being able to influence the behavior of individuals or groups [9]. Ensuring that the researcher is the same gender as the research participant is one way of potentially making more equal the power between researcher and participant, particularly for female research participants discussing sensitive health issues.

In this article, we report new insights about conducting sexual health research in PNG. A diverse nation made up of over 800 distinctly different language and cultural groups, PNG has corresponding diversity of beliefs and practices. However the majority of cultural groups are organised along patrilineal lines. Gender is thus a key dimension of social life that influences the nature of individual and collective relationships [10]. In PNG, gender refers to the characteristics assigned to women and men by the society in which they live [11]. Gender influences the type of tasks undertaken by men and women, employment opportunities and can indicate vulnerability to inter-personal violence and HIV [12–15]. Matching the gender of the researcher and research participant therefore reflects social and cultural patterns [16].

Taking into account the colonial history between researcher and participant is also critical to understanding potential power differences. As researchers from PNG, based in PNG, or Australians working in PNG, we are committed to enacting decolonising research methodologies. This is particularly important given that PNG gained independence from Australia in 1975. One key step towards decolonising research and addressing potential power imbalance between researcher and participants is for those of us who were formerly ‘the researched’ to become ‘the researchers’. Linda Tuhiwai Smith explains, “When indigenous people become the researchers and not merely the researched, the activity of research is transformed. Questions are framed differently, priorities are ranked differently, problems are defined differently, people participate on different terms” [6].

Researchers who enact decolonising methodologies prioritise power redistribution and acknowledge the reality of colonisation upon the research process [17]. Decolonising research rejects the notion that Western ideologies and worldviews are superior. They privilege Indigenous ways of knowing and understanding history. As observed by Kovach, decolonising research provides space for a shift for non-Indigenous researchers to move beyond the them/us binary and move towards “new, mutual forms of dialogue, research, theory, and action” [18]. It is with a commitment to respectful research practice that we critically reflect upon an experience of conducting qualitative interviews for HIV research, which challenged our assumptions and led us to learn more about how to conduct public health research in PNG.

## Methods

### Our experience of co-interviewing

In 2012, Unia Api (UA) a Papua New Guinean male from a university in PNG and Michelle Redman-MacLaren (MRM) a White Australian female from a university in Australia, conducted eight semi-structured interviews about HIV services with staff at a faith-based organisation in Lae, a large industrial city in PNG. The interviews formed part of a collaborative study undertaken to understand the influence of donors and the church on the faith-based organisation and how this influenced HIV prevention activities and treatment services [19]. The co-principal investigator of the study was the Late Pastor Matupit Darius (MD), a senior and highly respected church leader. MD was renowned across PNG and the Pacific as a film maker, actor and HIV advocate as well as being a theologian and academic at Pacific Adventist University. MD led UA and MRM to Lae in September 2012 and introduced UA and MRM to key church and faith-based organisation leaders. He also provided supervision and support for the two researchers conducting field work. Eight individual interviews were conducted in three sites across the city. Four of these interviews were conducted consistent with the planned gendered approach to individual interviews. However, four of the research participants (two managers - one male and one female and two HIV counsellors - one male and one female) requested to be interviewed by both UA and MRM. On each occasion, UA and MRM reiterated the offer to interview the two men and two women separately using the gendered approach planned. However, it was the expressed wish of each participant that both UA and MRM conduct the interview. The interviews progressed on this basis, with UA being lead interviewer during three interviews and MRM being lead interviewer during one interview. The lead interviewer asked the semi-structured questions prepared prior to the interview and the co-interviewer took notes, probed and ask supplementary questions. In addition the co-interviewer summarised the main points at the end of each of the interview. All interviews were voice recorded and transcribed verbatim.

When the interviews were concluded, the field team (UA, MRM and MD) critically reflected upon the co-interviews. We considered what influence our respective cultural heritage may have had on the interview process and content discussed, including how the ‘mismatched’ gender might have impacted the interview process. Our initial and primary concern was the fact two interviewers had actively co-facilitated the interviews with a single participant. Had co-interviewing increased the researchers’ power in the interview process and did this influence participant response? Conversely had co-interviewing increased the participants’ power in the interview process and correspondingly influenced their response?

### Ethics and adherence to qualitative research review guidelines

Ethical approval for the study was obtained from James Cook University Human Research Ethics Committee (H4295), Pacific Adventist University (Approval Letter, 10 March 2012) and the PNG National AIDS Council Secretariat (RES10 COM005). All participants gave their informed consent to participate in the study. Authors of this article confirm adherence to the qualitative research review guidelines and have carefully considered the relevance of the study question, appropriateness of qualitative method, transparency of procedures and soundness of interpretation.

### Insider-outsider research

Drawing upon the work of Louis and Bartunek [20], Ritchie and colleagues describe insider researchers as those who have had a place in the group being studied prior to the research starting [21]. Outsider researchers are described as those who begin to relate to the research topic only as the research study begins. Researcher experience is influential in shaping the interview process [22] and having a researcher who is identified as an insider can enhance the research process in decolonising research contexts [8]. To contextualise our findings and the subsequent discussion, we explicate the insider/outsider roles held by the two researchers. UA is a man from PNG who works at the “*ples blong save*” (‘place of knowledge’ - the University) in the same faith tradition as the faith-based organisation. He is also a *wantok* (literally, speaks the same language) of some of the research participants. MRM is as an Australian who speaks *Tok Pisin* (demonstrating extended links to Melanesia/PNG), has previously lived in PNG, had previously conducted HIV work with the faith-based organisation and was once a member of the faith tradition. Both UA and MRM hold an insider-outsider role, although in different ways.

## Results

### What we discovered

From the experience described above, we critically reflected on the advantages and disadvantages for the research participants to be co-interviewed by two people, a male from PNG and a female from Australia. We discussed how culturally-shaped concepts of audience, status and cultural safety could have contributed to the participants’ desire for two interviewers.

In the PNG context, a possible advantage for the research participant of having two interviewers is that two people provide greater audience. Greater audience could be seen to reflect an important status and elevate the contribution of the research participant. In the oral, *bikman* culture that is predominant in most of PNG, somebody who has a high status would expect to have as many people listening to his (or less commonly, her) opinions as possible. Having two interviewers therefore gave additional status to the study participant. Further, the audience provided by two interviewers, might have been seen as a way of ensuring ideas were communicated to the leader of the study, MD, who was a highly respected *bikman* in PNG. In addition, the research participants were able to speak to an international researcher and share their opinions, while still having somebody from PNG there to contextualise the knowledge generated during the interview. These dynamics may have enhanced the perception of both audience and potential influence over research outcomes that could benefit the people the research participants work with.

Being co-interviewed by two researchers may have been seen to enhance the value of the research participant’s contribution to influence their organisation. One participant explicitly stated that being involved in the research project gave an opportunity to speak out about areas of concern. “*As I’ve made reference earlier to the [name] church, research is the key so people like you, research practitioners like you… have a pivotal role because based on the research we only create activities that supports, that incorporate stories that you guys have already done*” (Research participant No.2). Being involved in the study was a way participants could advocate for people living with HIV and gain the support of their organisation.

It was our experience that co-interviewing enabled probing and additional questions not explored by the lead interviewer. This provided space for the research participant to provide more details about initial responses and enabled a rich co-generation of data that would have been more difficult to achieve with one interviewer. From the perspective of the researchers, the nature of the questions, the language used and meaning co-construction was richer than if there had only been one interviewer. However, for two researchers to conduct an interview with one research participant, whatever the gender or cultural backgrounds, the possibility exists of unequal power distribution in favour of the interviewers. This was not an apparent concern for the four research participants who actively invited both researchers to co-interview. These research participants were genuine in their requests to be co-interviewed, despite repeated offers of individual interviews by either researcher. This dynamic led us to review cultural understandings of the ‘interview’ and other researcher experiences in PNG and across Melanesia.

## Discussion

### Co-interviewing

Co-interviewing has been described as a method to bridge cultural and gendered divides [7, 23]. However, in sexual health research the interviewer explores highly sensitive topics such as relationships, sexual experiences and sexually transmitted infections. Nevertheless, it was the observation of both UA and MRM, as experienced researchers, that the research participants genuinely wanted us both to be there. In this professional context, where participants were employees of a faith-based organisation, having male and female interviewers as an audience of two from two different universities appeared desirable. Did the presence of both researchers further honour participants’ response to HIV in PNG, such as their personal experiences, the HIV prevention services, mainstreaming of HIV or attempts to address organisational impediments to undertake ‘culturally acceptable HIV activities’? In addition to discussing their professional response to HIV, some participants also talked about their own sexual health issues and measures taken to improve sexual health outcomes. Participants explicitly stated their desire to use results from the study as evidence to enhance service delivery and increase access to resources. As an example, a HIV program manager expressed concern that inadequate infrastructure was restricting the types of STI and HIV tests able to be provided at a clinic. This was included in the summary of results and presented to the faith-based organisation. Subsequent to this, infrastructure was improved and testing facilities are now available at the clinic. This exemplifies how power was understood and utilised by participants to achieve transformation within their realm of influence.

In the cultural context of PNG, co-interviewing increased the size and status of the ‘audience’, which correspondingly increased the status and possible outcomes of both the participant and their organisation (and by association the people they provide services for). It is also possible that co-interviewing involving two researchers was a mechanism to redistribute power away from a single interviewer who, when alone, had the power to influence what was reported. A strident criticism of some research, particularly in colonial or post-colonial situations, is that researchers have used ‘power-over’, rather than ‘power- with’ research participants or populations [24]. Use of ‘power-over’ by researchers perpetuates unjust power imbalances in the research process [6]. Co-interviewing provided a more culturally accountable process and diminished the power of an individual researcher to influence what was reported.

Our study used specific methods to devolve, share and enable the participant to have more power, consistent with our commitment to participative, decolonising research methods [6, 14, 18, 25, 26]. Methods employed included ensuring a private location for the interview and paying careful attention to the physical arrangements of the interview, including seating positions and room arrangement. Researchers also explicitly stated that the research participant was the expert in the interview and used open-ended qualitative questions to encourage the participant to discuss content that was most important to them [27]. These discussions were facilitated in *Tok Pisin* (a lingua franca of PNG), English (the language of the ex-colonisers) or a mixture of the two [28]. In PNG, there are three national languages *Hiri Motu, Tok Pisin* and English. Papuan peoples in the Southern region of PNG speak *Hiri Motu,* with *Tok Pisin* spoken throughout much of the remainder of the country. English is mostly used in formal education and for more official communication. Providing the option to discuss HIV in *Tok Pisin*, a language closer to the lived experience of most participants, provided another way to reduce potential power imbalances between researcher and participant.

In addition to physical surroundings and languages used, we also explained the reporting and feedback process and assured participant’s an opportunity to provide comments on preliminary findings prior to findings being reported publically (conducted in Lae during May 2013). Researchers and participants also discussed the potential to use results to advocate for greater resources. The risk was the presence of two researchers’ would inadvertently increase expectations about the study team’s ability to influence action based on information shared in the interview. Thus the onus was on the researchers to carefully and honestly explain the research process, who would receive the findings and who would be responsible for research-informed action.

### Co-production of knowledge

As a result of our critical reflection, we realise the study was bringing assumptions about power imbalance in research to a group of people from PNG with an indigenous worldview who perhaps conceived of power differently. In decolonising and indigenous research methods, power is understood to be held by the non-indigenous researcher who has come into the indigenous cultural context. In fact, research itself is seen as one of the “dirtiest words”, due to the misuse of power by non-indigenous researchers [6]. We have questions about the construction of researcher power in PNG, as described in some decolonising research theory.

Melanesian cultures are based on collective rather than individualist ideologies. Collectivist foundations inform practice, including how knowledge is produced/co-produced. It is therefore unusual for people to talk privately (one-on-one) unless they have a very close or intimate relationship. In fact, there are a number of risks inherent in agreeing to speak to someone privately. In a collectivist, oral tradition, if one speaks privately to another, there is no-one present to verify the truth of the assertions made. Talking privately may endanger the interviewee or interviewer if the partners or family members do not agree to the interview. In fact, as evidenced by the village courts in PNG, many witnesses to the evidence provided is often preferred - many people want to listen to, and verify, what is being said, including spouses, aunties, uncles and *bubus* (grandparents/grandchildren) [29]. Applying this cultural understanding, we have learnt that the way a decolonising researcher might seek to ethically ‘do no harm’ by conducting a private interview with a person of the same gender may, on some occasions, need to be enacted differently in Melanesia. The different sites of power remain, including positional power, relational power between workers and supervisors and formal and informal power in the interview context. However, the way this power is conceptualized and experienced by the research participant may be different. More power may be given to a participant by increasing or changing the researcher ‘audience’, for example by having two interviewers. This is consistent with cultural approaches that utilise public forums rather than private or more individualist/formal processes (for example, written submissions) to raise issues. Knowledge production and transmission is a site of powered interaction and requires a nuanced understanding of the two-way, collective, culturally constructed process that has specific and different characteristics based on Melanesian worldviews [30]. In the remainder of the paper, we reflect further on some key issues that have emerged as a result of critical reflection upon our research practice.

### Legitimacy in the research process

In Melanesia, everyone has their place [30]. Older people and senior leaders give legitimacy to the process of knowledge production and transmission. In this experience, the interviewers UA and MRM were afforded additional legitimacy by having MD, an esteemed senior church leader and HIV advocate, theologian and researcher help arrange the interviews. MD helped to organise the interviews and give his authority to the research project. UA and MRM were conducting the interviews, but people may have been more willing to participate in the study because MD was on location and supporting the research. The interviewers’ legitimacy arose from the respect afforded a more senior person in the social, cultural and religious context. It is our experience that when conducting research or training in PNG, it is important to have an older person and younger person together, so when the younger person conducts the interviews, or enacts their training (particularly in their village setting) they have a legitimacy to act. This is consistent with the experience of other researchers in PNG [31]. A senior researcher provides authority and status to the younger researcher’s activity. This was certainly the case with UA and MRM working under the authority and status of MD.

### Opportunity to speak

Melanesian culture has specific conditions that determine who can speak and who cannot [32]. Opportunities to be listened to depend upon a person’s status in the community. This dynamic also occurs in religious communities in PNG, where leaders of the Christian churches typically preach to people, rather than ask questions of people. Interviewees may have seen the invitation to participate in research conducted by a Christian University as a new approach - that the church leaders from the church university have come to my door. Researchers act differently to some church leaders when they provide a safe and non-judgmental environment and really listen. By saying *taem blong yu long toktok* (this is your time to talk) and carefully listening to interviewee responses, researchers devolve positional (and in some cases gendered and/or cultural) power and enable participants to articulate their concerns and opinions.

### Limitations

There are a number of limitations to the analysis of our co-interviewing experience. Only a small number of interviews were conducted (n = 8) and of these only half (n = 4) requested to be co-interviewed. However, this dynamic was consistent enough for us to identify a pattern that we had not expected in the original qualitative research design. We responded with flexibility and cultural sensitivity. There are also questions about why the other four participants did not request to be co-interviewed. Two of these four participants were employees of the faith-based organisation while two were employees of the church. Were we as researchers seemingly less available to offer this option, or did the participants genuinely prefer to be interviewed by one researcher? Did the cultural status or organisational status of the participants influence this outcome? Despite not knowing the answer to these questions, this experience has expanded our sensitivity to culturally safe and appropriate ways of conducting research in PNG and across Melanesia.

### Conclusions

As researchers, we are very committed to conducting research in an ethical manner and working in ways which are highly sensitive to the gender, social, and cultural values held by research participants [2, 33–36]. These values also reflect our understanding of power held by the researcher/s and the research participants. We know insider-outsider status can impact the research process. There are also cultural beliefs and practices that shape qualitative research and if deliberately employed, may help to address power imbalances. Culturally there may be more status provided to the participant when two researchers are present at the interview as greater import is placed on the content shared.

Reflexivity in research practice requires researcher/s to critically reflect upon the experience and position they bring, the methods they use and how they adapt research practice to incorporate learning [37]. A reflexive and critical approach to professional research practice questions how knowledge is generated and, further, how relations of power influence the processes of knowledge generation [38]. Qualitative research methods are constantly expanding as researchers with diverse worldviews participate in, lead and evolve culturally-relevant research practice [39]. Our recent experience has taught us to critically reflect upon accepted qualitative research methods and to centralise the cultural context in which research is being conducted. Research methods that are gender sensitive, culturally situated and relevant to the research question are pivotal to successful research in Melanesia and may challenge accepted ethical notions of ‘do no harm’ [31]. Successful research that is sensitive to gender and culture requires cultural knowledge, continuous critical reflection and researcher flexibility based upon respect.
